# Feasibility study of a newly developed solid gel pad containing tamarind seed gum for diagnostic ultrasonography in human subjects

**DOI:** 10.1038/s41598-025-33208-y

**Published:** 2026-01-12

**Authors:** Takuya Uehara, Hajime Monzen, Megumi Ujifuku, Yukinori Matsuo, Yutaka Watanabe

**Affiliations:** 1 Department of Radiation Oncology, Kindai University Faculty of Medicine, Osaka, Japan; 2 Department of Medical Physics, Graduate School of Medical Sciences, Kindai University, 1-14-1 Mihara-dai, Minami-ku, Sakai, Osaka, 590-0197 Japan; 3 Watanabe Kosei Clinic, Shiga, Japan

**Keywords:** Ultrasound diagnosis, Ultrasound gel, Diagnostic imaging, Tamarind seed gum, Health care, Medical research

## Abstract

Ultrasonography-based diagnosis is increasingly used owing to its reliability. However, the conventional ultrasound gel has limitations of patient discomfort and cost. This study evaluated the clinical utility of a newly developed gel pad for ultrasound diagnosis composed of tamarind seed gum (0.1–5.0 wt%), polyhydric alcohol (25.0–70.0 wt%), and water (30.0–70.0 wt%). Ultrasound imaging was performed in four healthy volunteers using our new solid gel and the conventional liquid gel. Linear, convex, and sector probes were used for imaging of the common carotid artery and thyroid gland, liver, and parasternal four-chamber view, respectively. Image quality and volunteers’ satisfaction were assessed using the 5-point Likert scale. For all sites, no significant differences in the image quality parameters were observed between our solid and the conventional liquid gels. The mean satisfaction score was significantly higher for all sites when our solid gel was used. Image quality remained unchanged for all sites after the application of our solid gel. The quality of images acquired using the new gel was comparable to that of those acquired using the conventional gel. This preliminary evidence supports the use of our new solid gel for ultrasonography with all probes for various tissue types and depths.

## Introduction

Ultrasonography (US) is a less invasive and more consistent imaging modality for assessing various organs in the body. It enables real-time visual characterization, and it has widely been adopted for diagnosis in various fields because of its acceptability and reliability.

US requires a gel or liquid medium to transmit waves from the probe to the organ of interest to produce images. The gel removes the air gap between the probe and skin surface, which results in improved image resolution and accurate interpretation of anatomy, physiology, and diagnosis^1^.

The conventional, commercial liquid US gel is the current gold standard because it is easy to apply, facilitates probe sliding, and is generally safe for patients and US equipment. However, it has several limitations, including patient dissatisfaction and cost^2^. Patients undergoing US examinations experience discomfort due to the application of the gel on their skin or hair, and the characteristic smell of the gel on the skin also affects providers performing US. Pre-warming the gel before examination and cleaning it from the skin of patients incur extra costs and are not cost-effective. The quantity of conventional gel used also varies with the providers performing US and the type of examination.

Furthermore, the conventional liquid gel dries out after approximately 15 min, and solid gel pads are recommended as alternatives^3^. The solid gel pad with gelatin was developed and validated in the previous study^3^. It remained undamaged after a 15-minute investigation, whereas the liquid gel dried out^3^. However, Kim et al. reported that the quality of the solid gel pad with gelatin, which was stored under strict temperature of 1–3 °C in a refrigerator and humidity control at 80% humidity, could be maintained for only approximately nine days^3^. In addition, Kim et al. described that short-term storage at room temperature (about 20–25 °C, about 12 days) required special attention as cracking of the surface might occur in the solid gel pad with gelatin^3^.

We developed a new solid gel pad with tamarind seed gum and a self-moisturizing property to solve these issues while maintaining the accuracy of US-based diagnoses. This study aimed to evaluate the clinical utility of our newly developed gel pad for US diagnosis.

## Methods

### Physical characterization of gel composition

#### Gel composition and molecular characteristics

The solid gel pad was manufactured with standardized dimensions of 50 × 50 × 5 mm (length × width × thickness). The gel comprised tamarind seed gum (0.1–5.0 wt%), polyhydric alcohol (25.0–70.0 wt%), and water (30.0–70.0 wt%). The tamarind seed gum, a natural polysaccharide extracted from tamarind seeds, has a molecular weight range of 50,000–1,000,000 Da and consists of a glucose backbone with β-1,4 linkages and xylose side chains. The polyhydric alcohol contained glycerin as the primary component (≥ 80 wt% of total polyhydric alcohols) and other polyols, including butanediol, propanediol, diglycerin, xylitol, and trehalose. Preservatives (parahydroxybenzoic acid esters) were incorporated to prevent microbial growth.

#### Rheological properties

Dynamic viscoelastic measurements were performed using a rotational rheometer equipped with 25-mm parallel plates (gap: 1.0 mm; sample volume: 1.0 mL). The storage modulus (G’) and loss modulus (G”) were measured in triplicate (*n* = 3) at 10°C, 25°C, 36°C, and 50°C. The measurement condition was as follows: frequency sweep from 0.1 to 100 Hz at a strain amplitude of 1%. The G’ was greater than the G” across all measured frequencies and temperatures, confirming its gel-like behavior and thermal stability suitable for clinical applications. This thermal stability ensures consistent acoustic transmission properties under varying clinical temperature conditions.

#### Mechanical properties

The compressive elastic modulus was determined using a tabletop precision universal testing machine in accordance with JIS K6254^4^. The cylindrical specimens (5-mm thickness) were compressed at 10 mm/min (*n* = 5). The gel demonstrated a compressive elastic modulus of 0.0062 ± 0.0008 MPa (mean ± SD, compressed by 25%), indicating optimal flexibility for conforming to skin contours during ultrasound examination while maintaining structural integrity.

#### Syneresis characteristics

Syneresis was evaluated by placing 1.5 g of the gel on filter paper and sealing it in polyethylene bags (*n* = 3). The gel had controlled syneresis with approximately 25% fluid release over 1 h. Analysis revealed that the syneresis fluid contained glycerin, water, and preservative components, enabling the gel to fill microscopic air gaps at the skin-transducer interface. This controlled fluid release mechanism facilitates optimal acoustic coupling and enhances ultrasound image quality.

#### Self-Recovery properties

The gel surface temporarily dried under controlled environmental conditions (25 °C, 50% humidity). However, it was re-moistened due to continued syneresis after resealing for 24 h, demonstrating its unique self-recovery capability and functionality during extended clinical use. This self-recovery property extends the working time and reduces the need for additional gel application during prolonged examinations. This characteristic enables the gel to maintain its acoustic properties after temporary exposure to air and ensures consistent performance throughout the examination without compromising image quality.

#### Manufacturing Process Details

The solid gel pads were fabricated through a standardized mixing and molding process designed to ensure reproducibility and consistent gel quality. Tamarind seed gum powder was first dispersed in deionized water under continuous stirring until complete hydration was achieved. Polyhydric alcohols, primarily glycerine with minor polyols, were then gradually added while maintaining room temperature (20–25°C) and continuous agitation to obtain a homogeneous viscous mixture. Preservatives in trace concentrations (< 0.5 wt%) were incorporated during the final mixing stage to prevent microbial growth.

The homogeneous mixture was degassed under vacuum to eliminate entrapped air bubbles and poured into rectangular molds (50 × 50 × 5 mm). Gelation was achieved by controlled cooling to room temperature, followed by demolding and individual packaging in moisture-barrier pouches under ambient conditions (20–25°C). This process yielded transparent, flexible solid gels exhibiting uniform thickness and self-moisturizing characteristics suitable for ultrasound coupling applications.

### Data collection and analysis of ultrasound images

Four healthy volunteers underwent US imaging with either our new solid gel or the conventional liquid gel (Aquasonic 100, Parker Laboratories, Inc., Fairfield, NJ). Ultrasound images were collected from the four healthy male volunteers for subsequent analysis. All participants had a body mass index within the healthy range and no known anatomical abnormalities or relevant medical history. Each volunteer provided informed consent before participation, acknowledging the voluntary nature of the research, its procedures, and associated potential risks. This study was conducted in accordance with the Declaration of Helsinki (as revised in 2013) and approved by our institutional review board (approval number, 20250325).

Imaging of the common carotid artery and thyroid gland with a linear probe, liver with a convex probe, and parasternal four-chamber view with a sector probe were performed to evaluate the interpretability for different tissue types, depths, and probes. US was performed by the same operator (TU) using the Versana Active ultrasound system (GE Healthcare, Chicago, IL, USA) with convex probe multifrequency (3–5 MHz), with a linear probe (8–13 MHz) and the Xario 200 ultrasound machine (Canon Medical Systems, Otawara, Tochigi, Japan) with a 1.8–4.2 MHz sector probe and standard presets for the organ of interest. Gain and dynamic range were adjusted for the common carotid artery as 5 and 72, the thyroid gland as 0 and 75, the liver as 10 and 72, and the parasternal four-chamber view as 90 and 60, respectively. Time-gain compensation was optimized automatically.

Image quality was assessed in real-time during US and scored using the 5-point Likert scale based on the total impression of all study images. The response options were 1 (very poor interpreted), 2 (poor interpreted), 3 (acceptable), 4 (well interpreted), and 5 (very well interpreted). The image quality assessments were performed by one radiologist and two radiologic technologists. The 5-point Likert scales for image quality and satisfaction were based on established assessment criteria and had preliminary validation confirming good inter-observer agreement among the three evaluators. The new solid gel was removed from the moisture after the package was opened, and US was performed for each healthy volunteer. The volume of the conventional liquid gel was not restricted for obtaining the images.

Furthermore, the four healthy volunteers who underwent US were asked to rate their satisfaction with our new solid gel and the conventional liquid gel on a 5-point Likert scale (1, very uncomfortable; 2, uncomfortable; 3, acceptable; 4, not uncomfortable; and 5, not uncomfortable at all). In addition, the image quality of our new solid gel was evaluated over time. One of the healthy volunteers underwent US for the four sites mentioned above at 0, 15, 30, 45, and 60 min after the start of use of our new solid gel. The US operator, all reviewers, and all healthy volunteers were unblinded to the use of our new solid gel and the conventional liquid gel.

Mann–Whitney U test were used to identify differences in image quality and satisfaction assessment with the use of our new solid gel and the conventional liquid gel. All analyses were performed using JMP software (version 14.0.0; SAS Institute, Cary, NC, USA), and differences were considered statistically significant at *p* < 0.05.

Of note, our solid gel is designed for single-patient use during ultrasound investigation sessions, with a typical operational duration of approximately 15 to 30 min.

### Comparison test with commercial solid gel

Additionally, we conducted a comparative evaluation between our new solid gel and two commercially available solid gels (Echo Gel Pad, Yasojima, Kobe, Japan and Aquaflex gel pad, Parker Laboratories, Inc., Fairfield, NJ). This comparison included an assessment of the aforementioned mechanical properties and syneresis characteristics.

## Results

**Fig. 1:**
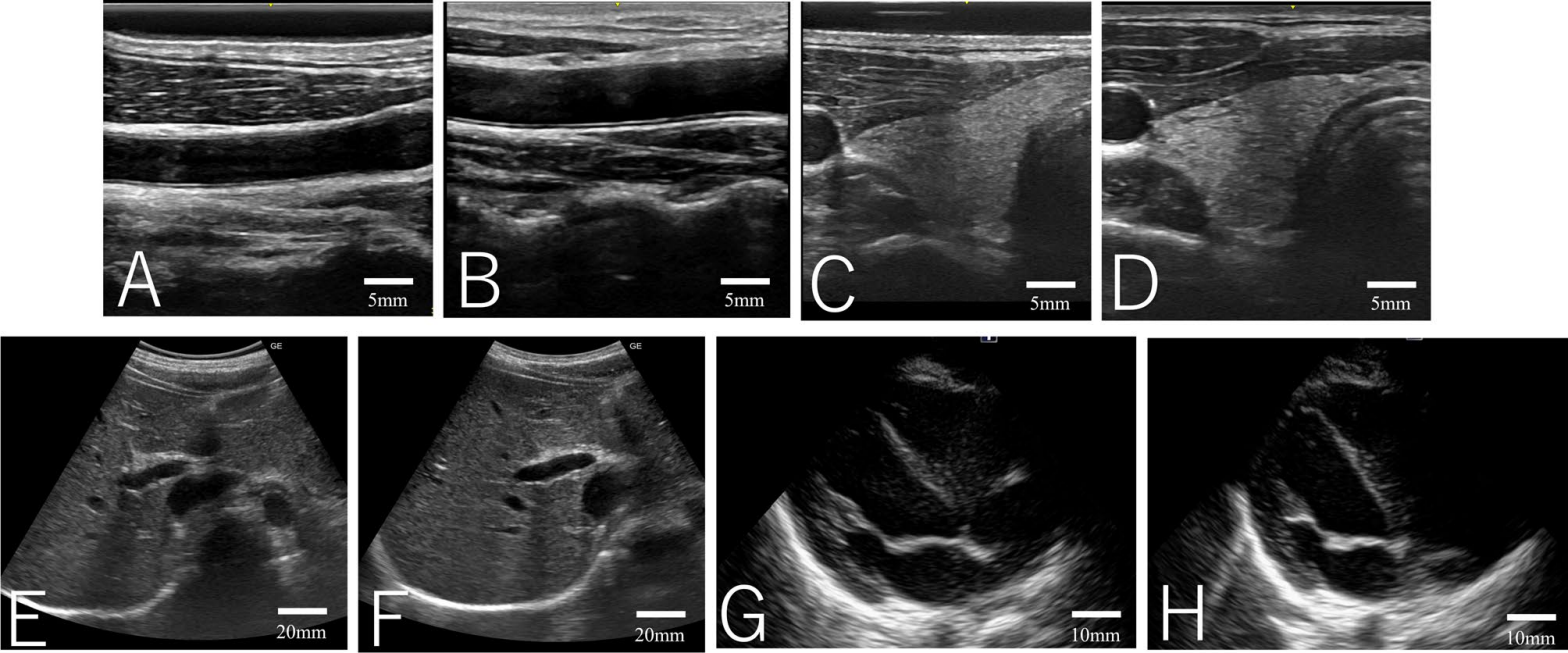
Ultrasound images obtained using our new solid gel and the conventional liquid gel. Common carotid artery: new solid gel (**A**) and the conventional liquid gel (**B**). Thyroid gland: new solid gel (**C**) and the conventional liquid gel (**D**). Liver: new solid gel (**E**) and the conventional liquid gel (**F**). Parasternal four-chamber view: new solid gel (**G**) and the conventional liquid gel (**H**).

The image quality parameters of our new solid gel and the conventional liquid gel are summarized in Table 1. No significant differences in the image quality parameters were observed with the use of our new solid gel and the conventional liquid gel for all sites. Figure 1 shows all sites for one of the healthy volunteers examined using our new solid gel and the conventional liquid gel.

**Table 1:** Image quality assessment with 5-point likert scale for ultrasonography with our new solid gel and the conventional liquid gel (*n* = 4).

	New solid gel (mean ± SD)	Conventional liquid gel (mean ± SD)	*p*-value
Common carotid artery	5.0 ± 0	5.0 ± 0	1.00
Thyroid gland	5.0 ± 0	5.0 ± 0	1.00
Liver	4.8 ± 0.3	4.7 ± 0.5	0.62
Parasternal four chamber view	3.7 ± 0.3	3.5 ± 0.3	0.40

Abbreviations: SD, standard deviation.

The volunteers’ satisfaction parameters are summarized in Table 2. The mean satisfaction scores for all sites were significantly higher with the use of our new solid gel than with the use of the conventional liquid gel (*p* = 0.02).

**Table 2:** Assessments of volunteers’ satisfaction using a 5-point likert scale for our new solid gel and the conventional liquid gel (*n* = 4).

	New solid gel (mean ± SD)	Conventional liquid gel (mean ± SD)	*p*-value
Common carotid artery	5.0 ± 0	1.9 ± 0.8	0.02
Thyroid gland	5.0 ± 0	1.9 ± 0.8	0.02
Liver	4.7 ± 0.5	2.4 ± 0.6	0.02
Parasternal four chamber view	4.7 ± 0.5	1.2 ± 0.5	0.02

Abbreviations: SD, standard deviation.

**Fig. 2:**
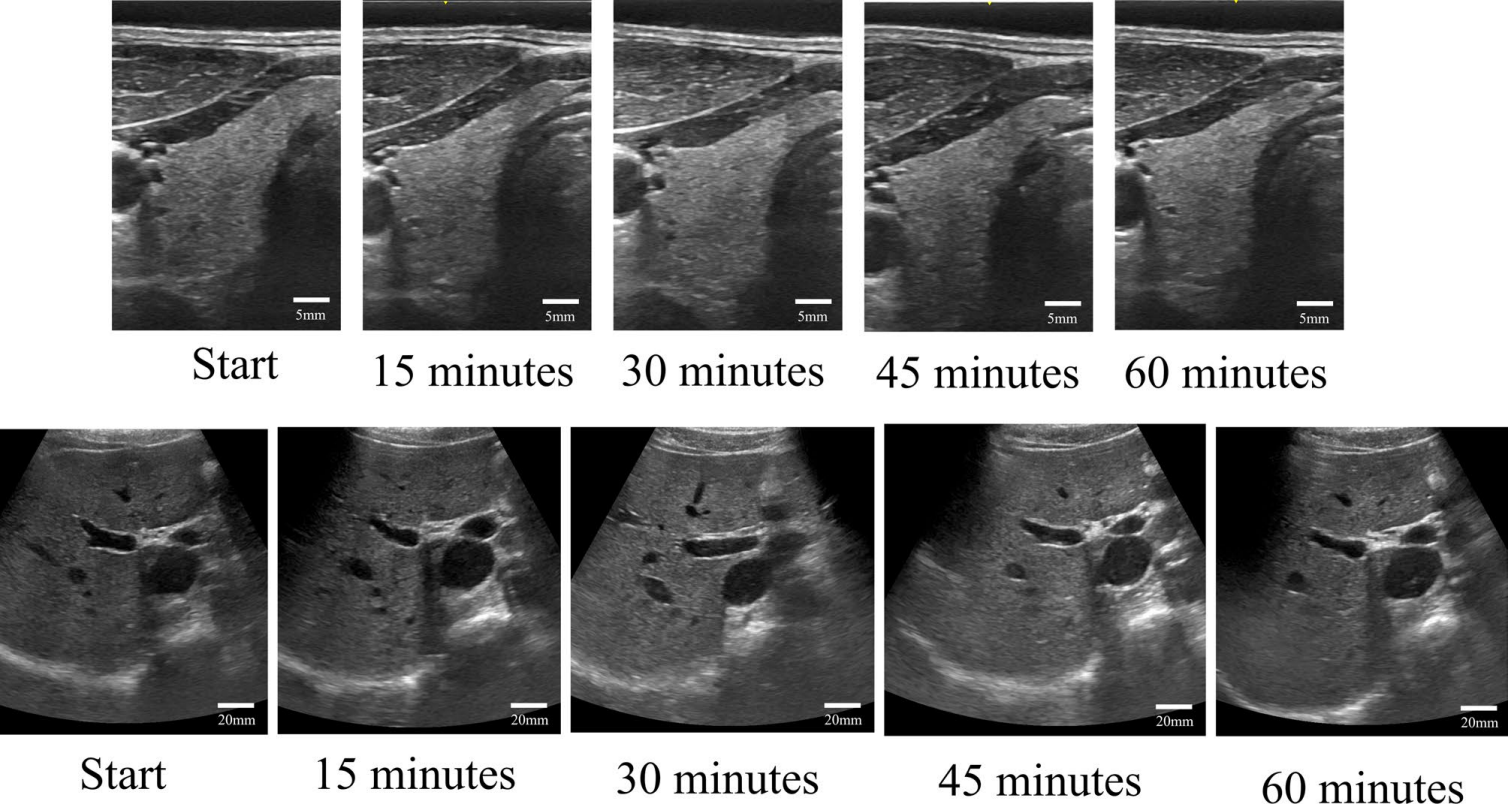
Images of thyroid gland and liver acquired at the start of investigation and 15, 30, 45, and 60 min later.

The image quality remained unchanged over time for all sites with the use of our new solid gel. Figure 2 shows the images of the thyroid gland and liver at the start of the investigation and at 15, 30, 45, and 60 min later. The drying tendency was not observed during the 60-minute investigation.

**Fig. 3:**
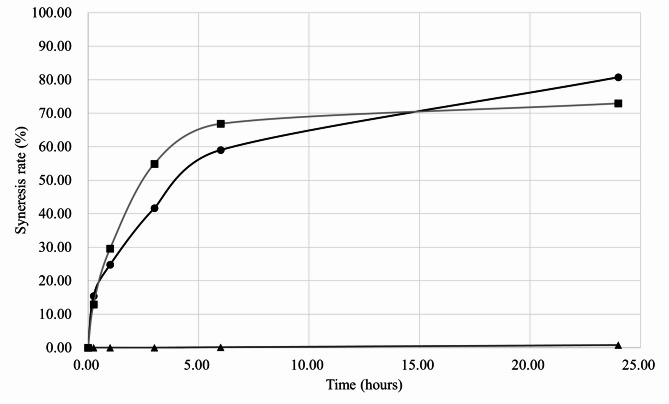
The syneresis rates from our new solid gel (line with circles), commercially available solid gel (Echo Gel Pad, Yasojima, Kobe, Japan) (line with triangles), and commercially available solid gel (Aquaflex gel pad, Parker Laboratories, Inc., Fairfield, NJ) (line with squares) were analyzed.

**Fig. 4:**
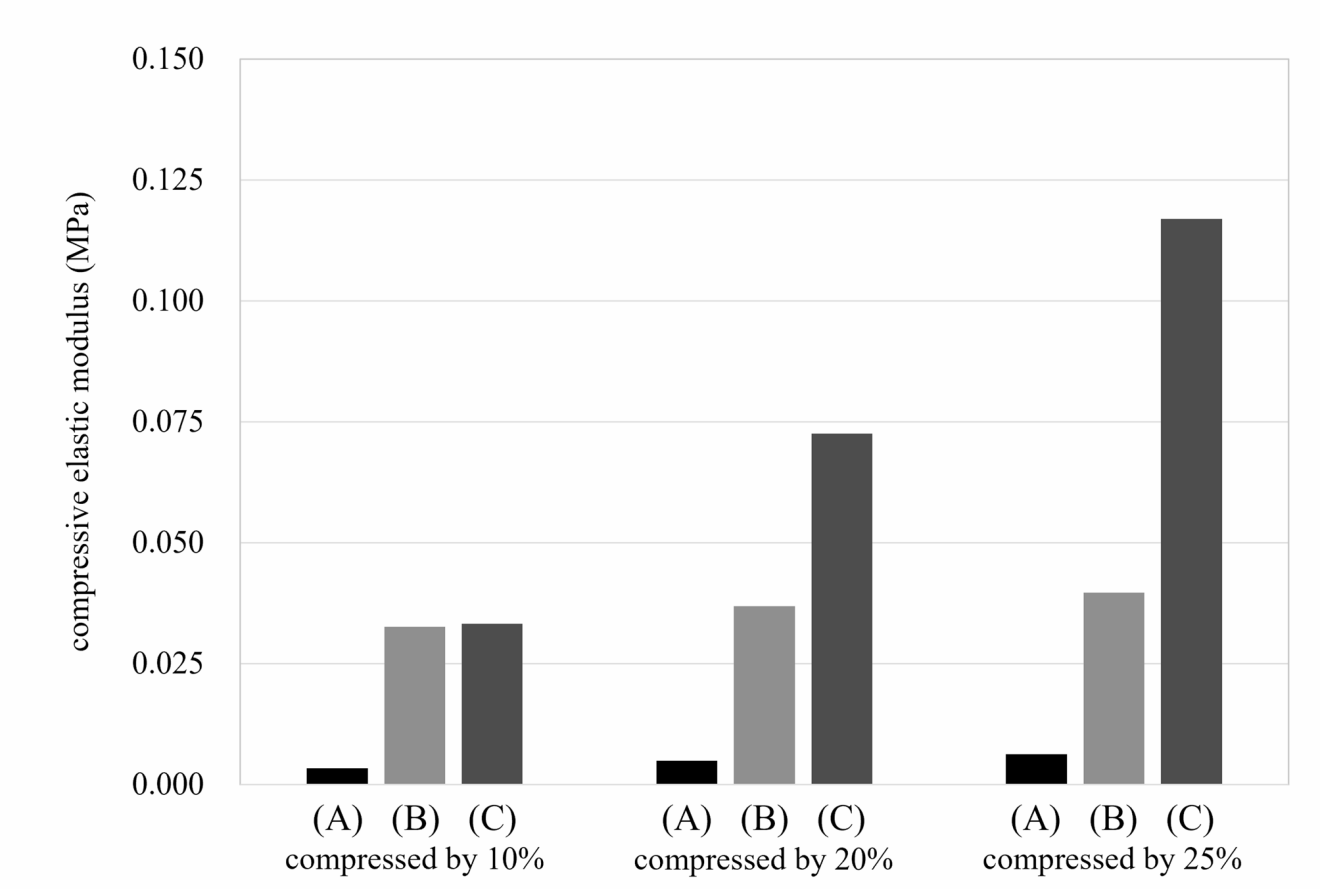
The compressive moduli of our new solid gel (**A**), commercially available solid gel (Echo Gel Pad, Yasojima, Kobe, Japan) (**B**), and commercially available solid gel (Aquaflex gel pad, Parker Laboratories, Inc., Fairfield, NJ) (**C**) were evaluated.

In addition, the syneresis rates and the compositions of the syneresed liquids from our new solid gel, commercially available solid gel B (Echo Gel Pad, Yasojima, Kobe, Japan), and commercially available solid gel C (Aquaflex gel pad, Parker Laboratories, Inc., Fairfield, NJ) were analyzed (Fig. 3). The analysis identified glycerin, water, and preservatives in the liquid exuded from our new solid gel, and glycerin and propylene glycol from solid gel C. In contrast, solid gel B did not exhibit syneresis. Each gel pad was resealed in a zippered polyethylene bag and allowed to stand at room temperature for 24 h. After this period, our new solid gel was observed to be re-moistened by syneresis fluid, while solid gels B and C remained dry. Furthermore, the compressive modulus of each gel pad was evaluated (Fig. 4). Compared to the two commercially available solid gels, our new solid gel demonstrated a lower compressive modulus.

## Discussion

The feasibility of ultrasound diagnosis using our newly developed solid gel with tamarind seed gum was evaluated in this study. Our study revealed that the image quality of our new solid gel was comparable to that of the conventional liquid gel during the US investigations of the common carotid artery and thyroid gland with a linear probe, liver with a convex probe, and parasternal four-chamber view with a sector probe. The US investigation using our new solid gel for all sites may be less uncomfortable than that using the conventional liquid gel. The US investigation over 60 min did not reveal any deterioration in image quality or drying.

The use of solid gel for US investigation is limited^3,5^. Kim et al. reported that they developed a solid gel pad with gelatin that remained undamaged after a 15-minute investigation. In contrast, the ultrasound liquid gel dried out^3^. However, the previous study evaluated the performance of the solid gel pad with gelatin during US using a phantom for only a sector probe. The performance of our new solid gel with tamarind seed gum was comparable to that of the conventional liquid gel in healthy volunteers, without image quality deterioration and drying out over time. To the best of our knowledge, this is the first study to report the clinical utility of our new solid gel with tamarind seed gum for ultrasound investigation with any probe on the body surface for variable tissue types and depths.

Krainin et al. demonstrated the tendency of increased patient satisfaction with heated ultrasound gel relative to the room-temperature gel^6^. In practice, patients who performed with the conventional liquid gel investigation reported discomfort due to its smell, texture, and temperature. Satisfaction with our new solid gel was evaluated in this study. The scores for satisfaction with our new solid gel were higher than those for the conventional liquid gel for all sites. This result may be attributed to the discomfort associated with the conventional liquid gel related to its adherence to chest hair and the difficulty of removal. The new solid gel, on the other hand, did not adhere to body hair and was easily removable.

Several alternatives to commercial acoustic gel have been reported in previous studies^5,7–9^. Previous studies have reported the need for an alternative ultrasound gel due to the unavailability and relatively high cost of commercial ultrasound gel in low-resource settings^8–11^. The key innovative component of our new solid gel is tamarind seed gum, a natural polysaccharide extracted from tamarind seeds. It provides the unique self-moisturizing function. This natural polysaccharide may be less expensive and easier to obtain than synthetic alternatives. Brailson Mansingh et al. demonstrated that tamarind seed polysaccharide is a natural polymer that is biocompatible and biodegradable^12^. Tamarind seed polysaccharide biopolymers extracted from the endosperm of tamarind seed have been used for controlled and targeted drug delivery applications by the pharmaceutical industry. In their review, Brailson Mansingh et al. described that almost all parts of the tamarind tree have found industrial applications and can contribute to the development of green and sustainable products^12^. Extra resources are often required to clean the conventional liquid gel from patients and probes after examination. The quantity of the conventional liquid gel used varies with the provider who performs the US and the type of examination. Our new solid gel can overcome these problems of extra costs due to unnecessary removal and stable usage. Kim et al. reported that the solid gel pad with gelatin was stored under strict temperature and humidity control, and its quality could only be maintained for a short period of approximately nine days^3^. However, our new solid gel has a self-moisturizing function and can therefore be stored at room temperature with resealing for extended periods. Accelerated stability testing at room temperature (20–25 °C) in sealed packaging for 3 months demonstrated no significant changes in rheological properties, syneresis, or ultrasound coupling performance. Comprehensive shelf-life validation studies are ongoing in accordance with International Council for Harmonisation of Technical Requirements for Pharmaceuticals for Human Use Q1A(R2) stability testing guidelines^13^. Specifically, samples are being stored and monitored under long-term conditions (25 ± 2 °C /60 ± 5% relative humidity).

The present study has several limitations. The sample was critically small (*n* = 4), and the anatomical sites were limited. The small sample size limits the statistical power and generalizability of the findings, particularly for the satisfaction assessments where statistical significance and large effect size were observed despite the limited sample size (Table 2). The results should be interpreted with caution, and further evaluation using larger cohorts and other anatomical regions with color Doppler and advanced imaging modes is needed for broader clinical application. Second, the study did not assess repeated use after disinfection or applicability to specialized ultrasound procedures such as extracorporeal shock wave lithotripsy and high–intensity-focused ultrasound. We thus propose use for single-patient or training applications only for diagnostic purposes, since cross-patient reuse currently conflicts with infection control standards. Third, the present study relied solely on subjective 5-point Likert scales and lacked objective image quality metrics, such as contrast resolution, signal-to-noise ratio, penetration depth, and lateral resolution. In addition, a simple phantom experiment comparing acoustic impedance measurements, attenuation, or insertion loss between using the conventional gel and the gel pad is also lacking in this study. For the results to be generalizable, future investigations should include standardized phantoms and quantitative analysis software so the results provide essential quantitative evidence beyond subjective image quality scores. Future studies should examine these limitations and explore the potential of the developed gel for point-of-care ultrasound applications across diverse clinical settings. Fourth, the inter-rater reliability among the three evaluators was not formally assessed, which may affect the reproducibility of the image quality scores. Formal intraclass correlation coefficient or Cohen’s kappa analysis is therefore needed in future larger-scale studies. Moreover, there was no blinded image evaluation, which might have introduced operator bias potential. Future studies should then include formal inter-rater reliability analyses and randomized crossover design with blinded image evaluation to strengthen the validity of subjective assessments. Fifth, beyond demonstrated technical performance, the path to market requires substantial focus on patient safety and economic viability. Subsequent studies with rigorous assessment of skin irritation and allergenic potential in accordance with ISO 10,993 endpoints as well as the generation of detailed biocompatibility profiles are therefore warranted. Concurrently, a robust economic evaluation, particularly a cost-comparison analysis encompassing production, storage, and disposal costs, is critical for enabling widespread clinical integration. Our preliminary economic assessment indicates that the solid gel pad can be manufactured and marketed at approximately 1,000 yen (~ 7 USD) per 50 × 50 × 5 mm pad for single-patient use. This production cost is economically competitive with that of the conventional liquid gel (200 mL bottle, ~ 300 yen or ~ 2 USD), considering a typical consumption of 10–30 mL per examination along with additional costs for pre-warming, cleaning, and variable usage patterns. This study provides a preliminary economic consideration for the use of the solid gel pad as a single-patient disposable product. The estimated unit manufacturing cost is approximately 1,000 yen per pad, which is higher than the direct per-use cost of conventional liquid gel. However, the total procedural cost may vary depending on workflow-related factors, including gel preparation, post-examination cleaning, and differences in gel usage volume among operators. Therefore, broader conclusions regarding cost advantages cannot yet be drawn, and a formal cost-effectiveness evaluation across clinical settings is required in future studies. Additionally, when comparing two commercially available gel pads, our new gel pad was observed to be re-moistened due to continued syneresis after resealing for 24 h, demonstrating its unique self-recovery capability and functionality during extended clinical use. However, these promising findings should be interpreted as part of a preliminary validation, and it would be premature to draw a generalized conclusion from these results alone. To fully establish the performance and practical advantages of our gel pad, further head-to-head testing against a wider range of commercial solid gel pads is warranted.

## Conclusion

The newly developed gel presented here has several distinctive properties optimized for ultrasound applications: thermal stability with consistent viscoelastic behavior across clinical temperature ranges, controlled syneresis enabling effective acoustic coupling, self-recovery through surface re-moistening that maintains acoustic properties even after air exposure, and optimal mechanical flexibility that balances structural integrity with skin conformability. These characteristics ensure reliable acoustic transmission, extended usability, and enhanced patient comfort during ultrasound examinations. The present feasibility pilot study was conducted as a preliminary investigation of the clinical utility of our new solid gel for US-based diagnosis across all probes for various tissue types and depths. Further research with a larger sample size is warranted to build up on this preliminary evidence.

## Data Availability

The datasets generated during and/or analyzed during the current study are available from the corresponding author on reasonable request.
